# Neurological complications and risk factors of cardiopulmonary failure of EV-A71-related hand, foot and mouth disease

**DOI:** 10.1038/srep23444

**Published:** 2016-03-22

**Authors:** Lili Long, Lin Xu, Zhenghui Xiao, Shixiong Hu, Ruping Luo, Hua Wang, Xiulan Lu, Zhiyue Xu, Xu Yao, Luo Zhou, Hongyu Long, Jiaoe Gong, Yanmin Song, Li Zhao, Kaiwei Luo, Mengqi Zhang, Li Feng, Liming Yang, Xiaoqi Sheng, Xuegong Fan, Bo Xiao

**Affiliations:** 1 Department of Neurology, Xiangya Hospital, Central South University, Changsha, 410008, China; 2 Department of Neurology, General Hospital, Tianjin Medical University, Tianjin, 300052, China; 3 Emergency center, Hunan Children’s Hospital, Changsha, 410007, China; 4 Hunan Provincial Center for Disease Control and Prevention, Changsha, 410005, China; 5 Department of Infectious disease, Hunan Children’s Hospital, Changsha, 410007, China; 6 Department of Genetics, The Maternity and Child Health Hospital of Hunan Province, Changsha, 410008, China; 7 Department of Intensive Care Unit, Hunan Children’s Hospital, Changsha, 410007, China; 8 Hunan Children’s Hospital, Changsha, 410007, China; 9 Department of Neurology, Hunan Children’s Hospital, Changsha, 410007, China; 10 Medical Records Management and Information Statistics Office, Xiangya Hospital, Central South University, Changsha, 410008, China; 11 The Maternity and Child Health Hospital of Hunan Province, 410008, China; 12 Department of Infectious disease, Xiangya Hospital, Central South University, Changsha, 410008, China

## Abstract

From 2010 to 2012, large outbreaks of EV-A71-related- hand foot and mouth disease (HFMD) occurred annually in China. Some cases had neurological complications and were closely associated with fatal cardiopulmonary collapse, but not all children with central nervous system (CNS) involvement demonstrated a poor prognosis. To identify which patients and which neurological complications are more likely to progress to cardiopulmonary failure, we retrospectively studied 1,125 paediatric inpatients diagnosed with EV-A71-related HFMD in Hunan province, including 1,017 cases with CNS involvement. These patients were divided into cardiopulmonary failure (976 people) group and group without cardiopulmonary failure (149 people). A logistic regression analysis was used to compare the clinical symptoms, laboratory test results, and neurological complications between these two groups. The most significant risk factors included young age, fever duration ≥3 days, coma, limb weakness, drowsiness and ANS involvement. Patients with brainstem encephalitis and more CNS-involved regions were more likely to progress to cardiopulmonary failure. These findings can help front-line clinicians rapidly and accurately determine patient prognosis, thus rationally distributing the limited medical resources and implementing interventions as early as possible.

Since the EV-A71 virus was first isolated in California, USA, in 1969^1^, it has become a threat to global public health. EV-A71 primarily infects children, and its clinical manifestations include hand, foot and mouth disease (HFMD) and herpangina. Some cases are accompanied by neurological complications, which manifest as brainstem encephalitis, aseptic meningitis, encephalitis, and acute flaccid paralysis (AFP), and some of the cases can progress to cardiopulmonary failure or even death^2^,^3^.

In the past 15 years, the Asia-Pacific region has witnessed several EV-A71 epidemics^4^,^5^,^6^. Nationwide epidemics in China began in 2008, with a total of 488,955 cases and 126 deaths while the population is 1328,020,000^7^,^8^. From 2010 to 2012, large nationwide outbreaks of HFMD occurred annually in mainland China, Hunan province in the central-south region of China was one of the hardest-hit regions. According to the data from the Hunan Provincial Center for Disease Control (CDC), there were 189,382 HFMD cases in Hunan in 2012, including 98 deaths while the population is 66,390,000^9^, and the mortality was ranked third among a total of 33 provinces around the country. Although an inactivated alum-adjuvant enterovirus 71 vaccine has completed Phase III clinical trials, it remains far from widespread application^10^,^11^,^12^. Thus far, the annual incidence, sequelae rate and mortality remain high in China, and the demand for medical resources exceeded the supply at the peak of the epidemic. According to the Hunan Provincial CDC, the incidence in May 2012 was 71.15/100000, and the Xinhua Net^13^ reported that approximately 500 clinical HFMD patients visited the Hunan Children’s Hospital daily. Although this hospital reserved all of the beds in wards and all emergency clinics to admit and treat paediatric patients with HFMD, it remained difficult to treat all of the pediatric patients seeking admission. Therefore, a challenge for front-line clinicians is to identify which paediatric patients are more likely to develop cardiopulmonary failure to rationally allocate limited medical resources and to intervene in cardiopulmonary failure as early as possible.

The Guide to the Clinical Management and Public Health Response for HFMD^14^ (hereafter referred to as the Guide) suggests that the clinical development of HFMD can be divided into four stages: simple HFMD, central nervous system (CNS) involvement, autonomic nervous system (ANS) dysfunction, and eventually, cardiopulmonary failure. CNS involvement is closely associated with severe sequelae and fatal cardiopulmonary collapse, but not all children with CNS involvement will demonstrate a poor prognosis. The results of a prospective clinical study during EV-A71 outbreaks in Sarawak showed that CNS complications were predicted by three risk factors (duration of fever ≥3 days, peak temperature ≥38.5 °C and a history of lethargy), but other early findings associated with severe or fatal disease have not been confirmed^15^. Therefore, it is very important for clinicians to rapidly determine which regions of the nervous system are affected and which neurological complications are more likely to lead to cardiopulmonary failure; these factors may predict the prognosis and prevent the progression as early as possible. However, reliable methods of predicting which patients with CNS involvement will develop into cardiopulmonary failure are still lacking^2^. The existing literature^6^,^14^,^16^,^17^,^18^,^19^,^20^ suggests that brainstem encephalitis may be a risk factor for cardiopulmonary failure, but the number of cases studied has been small, and the data have not been statistically analysed. Huang *et al.* classified myoclonus as the clinical manifestation of grade I brainstem encephalitis^20^, but most cases demonstrate only myoclonus and no other evidence of brainstem encephalitis^21^. Myoclonus may occur in normal young children, particularly when they are asleep^2^. The literature has also reported that myoclonus alone cannot be used as a diagnostic indicator of brainstem encephalitis^21^. Moreover, the involvement of any one of the following areas can cause myoclonus: the cerebral cortex, the brainstem, the cerebellum, and the spinal cord^22^. Therefore, in addition to the four neurological complications described by the Guide^14^, we also studied the association between EV-A71-related HFMD with simple myoclonus and cardiopulmonary failure.

In the present study, we analysed the medical records of 1,125 HFMD patients who were infected with EV-A71 and hospitalized in the Hunan Children’s Hospital (a Chinese provincial hospital designated for HFMD) during the period of 2010–2012, including 1,017 cases with CNS involvement, in an attempt to identify the possible predictive indicators of cardiopulmonary failure.

## Methods

Our study was granted approval from the ethics committees of Xiangya Hospital and the Hunan Children’s Hospital. In addition, we obtained informed consent from patients whose photographs were used in this manuscript. This study was conducted in accordance with the approved guidelines and regulations, in line with the tenets of the Declaration of Helsinki.

### Pathogen detection

Case specimens (throat swabs, stool samples, fluid from blisters and cerebrospinal fluid) of the patients were collected by the Hunan Children’s Hospital. Viral RNA extracted from samples submitted for diagnostic assays was performed using the Viral Nucleic Acid Extraction Kit II (Geneaid Biotech Ltd., Taiwan) according to the manufacturer’s instructions^23^. A diagnostic kit for CV-A16 and EV-A71 RNA (Fluorescent PCR) (Bioperfectus technologies, Jiangsu, China) was used to conduct simultaneous detection of human EV-A71 and CV-A16 by multiplex real-time PCR with an internal amplification control^24^. If any of the case specimens was positive, we can make the diagnosis of EV-A71 or CV-A16^5^.

### HFMD patients infected with EV-A71

Among the clinically diagnosed HFMD patients hospitalized in the Hunan Children’s Hospital from January 2010 to December 2012, 1,392 cases were diagnosed as EV-A71-related HFMD based on EV-A71 positivity detected by PCR test. Our study focused on EV-A71 positive HFMD. To maximize the specificity of our results, patients with EV-A71/CV-A16 (coxsackievirus type A 16) co-infections were excluded. After removing 267 cases with CV-A16/EV-A71 double-infection, 1,125 medical records remained for the present retrospective study.

### Case definition

The case definitions were based on the Guide^14^. HFMD was defined as a febrile illness with a typical rash on the mouth, palms and/or soles of the feet, which often occurs in infants and young children. HFMD patients with myoclonus alone included the presence of only myoclonus, without other neurological symptoms or signs. In our present study, there were 559 cases of myoclonus alone, which were more frequent compared with the previous normal period, indicating that the CNS was affected. However, we could not conclude which regions of the CNS were damaged because of myoclonus alone. Therefore, we thought of myoclonus alone as one type of the neurological complications. Neurological complications with or without myoclonuscan be divided into aseptic meningitis, brainstem encephalitis, encephalitis, and acute flaccid paralysis (AFP). Aseptic meningitis was defined as a febrile illness with headache, vomiting and meningism associated with more than 10 white cells/mm^3^ cerebrospinal (CSF) fluid and negative results of CSF bacterial culture. Encephalitis was defined as impaired consciousness, including lethargy, drowsiness, coma or seizures with or without myoclonus. Brainstem encephalitis (rhombencephalitis) was defined as any combination of ataxia, nystagmus, oculomotor palsies, and bulbar palsy, with or without myoclonus or an abnormal MRI. AFP was defined as the acute onset of flaccid muscle weakness and a lack of reflexes. AFP includes polio-like anterior horn cell involvement (anterior myelitis), Guillain-Barre syndrome (or peripheral nerve damage), and transverse myelitis^2^. To summarize all of the neurological complications, we defined mixed CNS involvement as any combination of the above four types of neurological complications. Definitions of ANS dysregulation, pulmonary oedema/haemorrhage and cardiorespiratory failure were accordant with the Guide^14^. Leucocytosis was defined as a white blood cell (WBC) count greater than 17,500 cells/μL on admission^26^, hyperglycaemia was defined as a blood glucose concentration greater than 8.3 mmol/L^26^, and intracranial hypertension was defined as a cerebrospinal fluid pressure greater than 180 mm H_2_O. The indicators for heart rate, respiration, blood pressure, and lymphocyte subset abnormalities are listed in Supplementary Tables S1 and S2.

### Definition of outcome

HFMD patients infected with EV-A71 were divided into cardiopulmonary failure group and group without cardiopulmonary failure. According to the Guide^14^, we defined cardiorespiratory failure as those with any of the following conditions: tachycardia, respiratory distress, pulmonary oedema, poor peripheral perfusion requiring inotropes, pulmonary congestion on chest radiography and reduced cardiac contractility on echocardiography. The dataset was randomly divided into two subsets: (1) the validation set (1,065 cases), and (2) the test set (60 cases). Patients were taken from the entire sample according to a conventional random number generation procedure and were then allocated to the two subsets with pre-determined sizes.

### Follow-up

The children were reviewed to assess neurological sequelae by a neurological examination undertaken by a trained doctor 1 year after discharge. At review, a modified Rankin Scale^27^ score was obtained to quantify disability (0 is normal, 1 is mild symptoms and no disability, 2 is slight disability, 3 is moderate disability and ability to walk independently, 4 is moderate severe disability and ability to walk with assistance, 5 is severe disability and inability to walk, and 6 is death).

### Statistical analysis

The data were analysed using SPSS statistical software (IBM version 19.0, Chicago, Illinois, USA). The continuous variables were analysed using Student’s t test, and the categorical variables were analysed using the χ^2^ test. When the expected values were less than 5, we used Fisher’s exact test for correction. All of the statistical tests were two-tailed, and a *P* value less than 0.05 was considered significant. The validation subset was for the regression analysis. All of the variables except age were binary, whereas the age was considered a categorical variable (0–1.5, 1.5–2, 2–3 years, and 3+ years) and was converted into a dummy variable. We used the 3+ years group as the reference group, X_1_ represented the 0–1.5 years group, X_2_ represented the 1.5–2 yeasr group, and X_3_ represented the 2–3 years group. First, we used a univariate logistic regression analysis to screen out the possible risk factors for cardiopulmonary failure from possible variables, and *P* values smaller than 0.2 were considered statistically significant. Second, a multiple logistic regression analysis was used to examine the multivariate-adjusted odds ratios for risk factors that were significant in the univariate analysis in which the backward stepwise elimination was used. Starting from the weakest predictor, variables were eliminated one by one from the full model until the change in R^2^ became statistically significant. *P* values smaller than 0.05 were considered statistically significant. Confounders were taken into consideration. Adjusted odds ratios (OR) and their 95% confidence intervals (95% CIs) were calculated. The Hosmer-Lemeshow test was used to examine the goodness-of-fit. Third, the regression model was built, the receiver operating characteristic curve (ROC curve) was plotted and the area under the curve (AUC) was calculated. We selected the *P* value as the cut-off when the Youden index was the maximum. Finally, the regression equation obtained was then applied to the test subset, and the sensitivity, specificity, positive predictive value (PPV), negative predictive value (NPV) and accuracy rate were calculated.

## Results

### Epidemiological information and clinical manifestations

**Figure 1:**
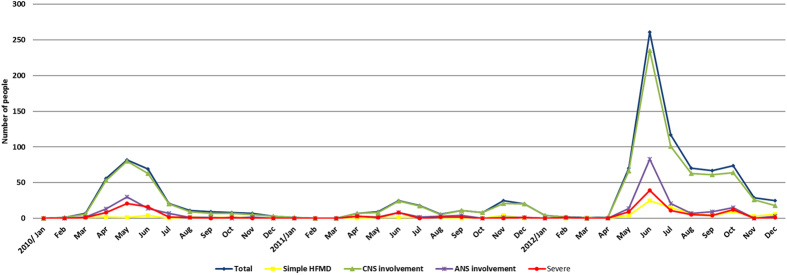
Number of Cases of EV-A71-related HFMD hospitalizations in the Children’s Hospital of Hunan province from January 1, 2010 to December 31, 2012.

The epidemiological data for the 1,125 patients are shown in Fig. 1. Most of the patients presented with fever (83.9% of the total number of patients), rashes (66.4%), oral ulcers (63.6%), and myoclonus (34.4%) upon disease onset. In addition, these patients also exhibited vomiting, headache, seizures, tachypnea, limb weakness or gait disturbance, irritability, drowsiness, and coma during the course of the disease. The 1,125 cases included 95 cases (8.4% of the total patients) of simple HFMD, 1,017 cases (90.4%) associated with CNS involvement (including some with ANS involvement and severe disease), 247 cases (22.0%) with ANS involvement (including some severe cases), and 149 patients (13.2%) with severe disease. The most common neurological complication was myoclonus alone (559 cases, representing 55.0% of the total neurological complications). The other neurological complications observed included encephalitis(289, 28.4%, alone and mixed), meningitis (253, 24.9%), brainstem encephalitis(158, 15.5%), and AFP (50, 4.9%). Detailed numbers of cases showing each type of neurological complication are listed in Table 1. Apart from myoclonus alone, the most common CNS complications during the epidemic season were nearly the same in each month; encephalitis or meningitis. The detailed numbers of cases showing each type of neurological complication in every month are listed in Supplementary Table S3. The average age of the patients with cardiopulmonary failure was younger than that of the simple HFMD patients (p = 0.026), and the average hospital stay of the patients with cardiopulmonary failure was approximately 2.6 times longer than that of the simple HFMD patients (Table 2). Because most patients were discharged after improvement or recovery (1,103, 98.0% of total), the average hospital stay could indirectly reflect the disease duration (Table 2). The average time (±SD) from the onset to CNS involvement was 2.05 (±1.63) days, the average time from CNS involvement to ANS involvement was 2.59 (±1.77) days, the average time from CNS involvement to cardiopulmonary failure was 2.63 (±1.84) days, and the average time from ANS involvement to progression to cardiopulmonary failure was 7.80 (±6.14) hours.

### Auxiliary examinations

**Figure 2:**
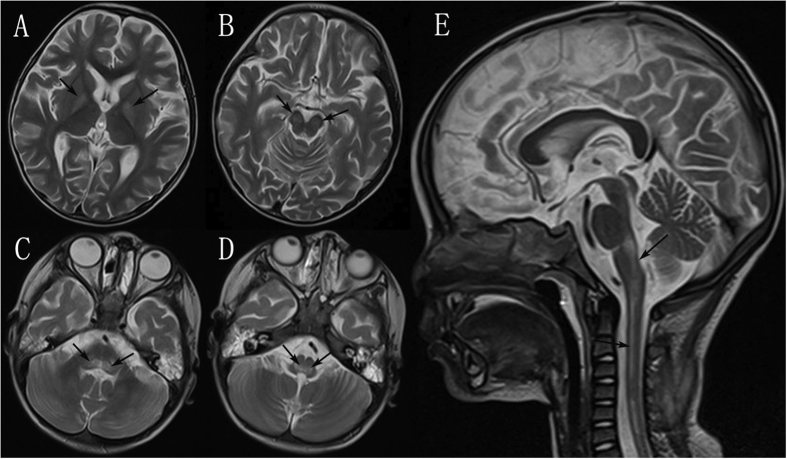
Spin-Echo T2-weighted MRI scan of a 28-month-old boy with encephalitis, brainstem encephalitis and myelitis from EV-A71 infection. The child had fever; rashes on the mouth, palms, and soles of the feet; vomiting; myoclonus; tachypnea; and drowsiness, followed by coma. Physical examination revealed skin rashes, weakness in four limbs, hypermyotonia, and positive reflex of Babinski’s sign. At the basal ganglia level (Panel **A**), there was an increased signal intensity of the bilateral globus pallidus (arrows). At the midbrain level (Panel **B**), there was an increased signal intensity of the bilateral cerebral peduncle (arrows). At the pons and medulla oblongata level (Panel **C,D**), lesions of high signal intensity were observed in the bilateral tegmentum (arrows). In the sagittal view (Panel **E**), there was a high signal intensity from the brain-stem to the anterior horn of the cervical spinal cord (arrows).

A total of 411 patients underwent chest X-ray examination, of which 263 cases were abnormal, and the main types of lesions included interstitial changes in both lungs, patchy shadows, pulmonary oedema and pulmonary haemorrhage. A total of 300 patients underwent magnetic resonance imaging (MRI) of the head or spinal cord within three days of entering the hospital (in the progressive stage of the symptoms, except for the patients who had been under endotracheal tubes since their arrival to the emergency unit and whose cardiac and pulmonary functions required closely monitoring), and 85 cases had abnormalities. The main manifestations were hyperintense signals on T2-phase images: 47 patients (accounting for 46.5% of the brainstem encephalitis patients who underwent MRI) demonstrated brainstem abnormalities (lesions involving the pontine tegmentum, medulla oblongata, midbrain and cerebellar dentate nuclei). Thirty-four cases (22.5%) showed brain abnormalities, with lesions involving the cortex, basal ganglia, globus pallidus, thalamus, hippocampus and corpus callosum, and seven cases (58.3%) had spinal cord abnormalities, with lesions mainly involving the anterior horn of the cervical or thoracic spinal cord (Fig. 2). In addition, 280 patients underwent electroencephalography (EEG) examination, of which 110 cases presented nonspecific slowing of background activity. Among these patients, three demonstrated sharp waves in certain brain areas (frontal and central) and seizures, and one of these patients had a history of epilepsy. Additionally, 40 patients had received electromyography (EMG), of which 21 cases showed neurogenic lesions.

### Relationships between various factors and cardiopulmonary failure

We selected 23 variables based on the literature and previous clinical experience (shown in Supplementary Table S4) to investigate the effects of these factors on the development of cardiopulmonary failure. We found that young age, fever duration ≥3days, tachypnea, tachycardia, hypertension, capillary refill time (CRT) >2 s, hyperglycaemia, leucocytosis, C-reactive protein (CRP) ≥40 mg/L, myoclonus, vomiting, coma, limb weakness, drowsiness and ANS involement were significant risk factors for cardiopulmonary failure.

We combined tachypnea, tachycardia, hypertension, capillary refill time (CRT) >2 s, and hyperglycaemia into one variable, referred to as ANS involvement, because these variables were all clinical manifestations or laboratory results implicating the ANS involvement and appeared on a single patient nearly simultaneously. We did not put leucocytosis and C-reactive protein (CRP) ≥40 mg/L, into the following multivariable logistic regression because not every patient underwent CRP and blood testing.

We then introduced age, fever duration ≥3 days, ANS involvement, myoclonus, vomiting, coma, limb weakness and drowsiness into a multivariate logistic regression model. We found that the respective risk of developing severe disease in the 0–1.5,1.5–2 and 2–3 years age groups were 9.3, (adjusted odds ratio = 9.3[3.6–23.8], p = 0.000), 5.0 (adjusted OR = 5.0[1.9–13.3], p = 0.001) and 5.6 times (adjusted OR = 5.6[2.1–15.0], p = 0.001) greater compared with the age group 3+ years. Additionally, we found that fever duration ≥3 days (adjusted OR = 9.4[1.4–62.5], p = 0.020), coma (adjusted OR = 27.7[5.3–145.4], p = 0.000), limb weakness (adjusted OR = 5.0[2.6–9.6], p = 0.000), drowsinesss (adjusted OR = 4.1[2.5–6.9], p = 0.000) and ANS involvement (adjusted OR = 13.5[8.2–22.3], p = 0.000) were the most significant predictive factors associated with cardiopulmonary failure (Shown in Table 3). The Hosmer-Lemeshow statistics indicated a non-significant lack-of-fit (χ^2^ = 6.496, p = 0.483).

**Figure 3:**
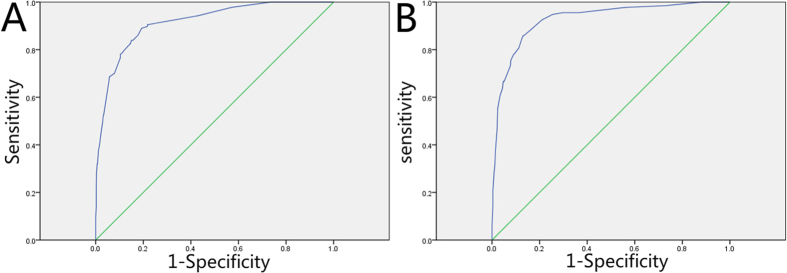
ROC curve of the final model. Panel **(A)** is the ROC of the first model, AUC = 0.916; 95% CI, 0.890–0.941, p = 0.000, Panel **(B)** is the ROC of the second model, AUC = 0.927; 95% CI, 0.903–0.952, p = 0.000, both panels indicate good internal validity.

We drew the ROC curve, which showed an acceptable performance on the validation subset (AUC = 0.916; 95% CI, 0.890–0.941, p = 0.000; Fig. 3, Panel A).

We applied the model in the test subsets (60 cases) to predict the probability of cardiopulmonary failure in HFMD. It had a sensitivity of 75.0%, a specificity of 81.3%, a PPV of 50.0%, and a NPV of 92.9%, and it correctly classified 80.0% of cases.

### Relationships between neurological complications and cardiopulmonary failure

We also investigated the effects of different neurological complications on disease severity. We collected data from 1,017 cases with neurological complications (excluding the simple HFMD cases in both the validation and test subsets). We found that myoclonus alone, encephalitis alone, and meningitis alone were significant protective factors, suggesting that EV-A71-related HFMD patients with only myoclonus, encephalitis or meningitis were less likely to develop cardiopulmonary failure compared with those with other neurological complications. By contrast, brainstem encephalitis alone, some combination of neurological complications (brainstem encephalitis combined with encephalitis, brainstem encephalitis combined with meningitis, brainstem encephalitis combined with AFP, encephalitis combined with AFP and meningitis combined with AFP), and the number of CNS-involved regions were significant risk factors for cardiopulmonary failure. Young age might be a confounder (the detailed unadjusted OR is shown in Supplementary Table S5).

We then introduced these 10 screened significant factors and age into a multivariate logistic regression model. After the confounding factor (age) was adjusted, we found that brainstem encephalitis alone (adjusted OR = 9.4[4.3–22.1], p = 0.000), brainstem encephalitis combined with encephalitis (adjusted OR = 10.5[4.2–26.0], p = 0.000), brainstem encephalitis combined with meningitis (adjusted OR = 2.3[1.0–5.2], p = 0.056), and the number of CNS-involved regions (adjusted OR = 6.3[4.6–8.6], p = 0.000) were the most significant factors associated with cardiopulmonary failure (Shown in Table 3). The Hosmer-Lemeshow statistics indicated a non-significant lack-of-fit (χ^2^ = 4.420, p = 0.620). Although there were six cases of myoclonus alone that developed into cardiopulmonary failure, they all had symptoms of ANS involvement.

We drew the ROC curve, which showed an acceptable performance on the validation subset (AUC = 0.927; 95% CI, 0.903–0.952, p = 0.000; Fig. 3, Panel B).

We applied the P_2_ model in the test subsets (57 cases with neurological complications) to predict the probability of cardiopulmonary failure in HFMD. It had a sensitivity of 58.3%, a specificity of 88.9%, a PPV of 58.3%, and a NPV of 88.9%, and it correctly classified 82.5% of cases.

The detail and the usage of the model was in the supplement.

### Treatment and prognosis

In the present study, 482 patients received gamma globulin treatment, 491 patients received high-dose methylprednisolone shock therapy, of which 387 patients combined methylprednisolone shock therapy with IVIG according to Chinese guideline for HFMD^25^,^28^,^29^,^30^, however, WHO guidelines do not recommend the use of corticosteroids in any severity of HFMD^14^. A total of nine patients died, and one patient withdrew from treatment during hospitalization. During the one year follow-up period after discharge, five patients died (three patients who required ventilator-assisted breathing died several days after discharge, one patient who had difficulty swallowing died two months after discharge and one patient with limb weakness and mental retardation choked while eating and died of suffocation one year after discharge). The mean age (±SD) of the deceased patients was 1.59 (±1.03) years old. In total, 72 patients (6.8%) presented sequelae, their mean MRS(±SD) was 1.96 (±1.79), including 65 patients (5.5%) with abnormal gaits or limb weakness, four patients required long-term assistance of mechanical ventilation, two patients experienced seizures, and one patient developed facial paralysis. The mean age (±SD) of the patients with sequelae was 1.78 (±0.98) years old. The sequelae and mortality rates were 7.1% and 1.5% for CNS involvement, respectively, whereas the sequelae and mortality rates were 33.6% and 9.4% for cardiopulmonary failure, respectively.

## Discussion

From January 2010 to December 2012 in the Hunan province, the major peak incidence of HFMD occurred each year from May to July, and a minor high peak occurred in November.

Among the viruses that cause HFMD, EV-A71 and CV-A16 were the most common, and EV-A71 was more likely to cause neurological complications and cardiopulmonary failure^31^. We did not find that CV-A16 could easily cause neurological complications or cardiopulmonary failure. Our cases confirmed that EV-A71 infection can cause the following neurological complications: encephalitis, meningitis, brainstem encephalitis, and AFP. The most common symptom associated with neurological complications was myoclonus; furthermore, encephalitis and meningitis were the most common neurological complications associated with EV-A71-related HFMD apart from simple myoclonus. MRI showed that the most frequently involved brainstem area was the pontine tegmentum, and the most frequently involved spinal cord area was the anterior horn; these findings are consistent with previous reports^17^,^20^,^32^,^33^.

Chen *et al.* analysed the EV71 epidemiological characteristics in Taiwan during a period of eight years and concluded that children of approximately 0.5–1 years of age demonstrated the highest mortality^34^. Our study also found that young age was a risk factor for cardiopulmonary failure, particularly 0–1.5 years old. Chang *et al.*^26^ reported that hyperglycaemia, leucocytosis, and limb weakness were the most significant risk factors for pulmonary oedema. Li *et al.*^35^ analysed the clinical characteristics of HFMD in China in a meta-analysis and concluded that blood glucose levels and WBC counts become elevated as the disease progresses. Fever duration, abnormal heart rate variability, vomiting, and impaired consciousness have also been reported to be correlated with cardiopulmonary failure^2^,^36^,^37^,^38^. In 2014, a mata- analysis was conducted regarding the risk factors for severe HFMD: this study found that a duration of fever ≥3 days, lethargy, hyperglycaemia, and young age were significantly related to the risk of severe HFMD^39^. Our data quantitatively confirmed the correlations between these risk factors and cardiopulmonary failure by univariate analysis. In addition, we found that tachypnea, hypertension, CRT >2 s, and CRP ≥40 mg/L were also risk factors; and among the above factors, our results revealed that young age, fever duration ≥3 days, coma, limb weakness, drowsiness and ANS involvement were more significantly associated with cardiopulmonary failure. Apart from age and fever duration, the above risk factors mainly focused on the ANS symptoms (hyperglycaemia, abnormal heart rate, tachypnea, hypertension, CRT >2 s) and CNS symptoms (limb weakness, vomiting, and impaired consciousness), ANS invovlement often develops into cardiopulmonary failure within several hours, and interventions to prohibit disease progression become difficult once this occurs. Therefore, ANS-related risk factors are not helpful clinical predictors of cardiopulmonary failure. Our results revealed that the mean time from onset to CNS involvement was 2.05 ± 1.63 days, and the mean time from CNS involvement to cardiopulmonary failure (2.63 ± 1.84 days) was generally more than 24 hours. The involvement of different part of the CNS invovled may lead to different outcomes; thus, in addition to observing CNS symptoms and signs, we should determine which regions of the CNS are affected by complications and which types of neurological complications have more predictive value to identify cardiopulmonary failure.

The Guide^14^ recommends that simple myoclonus and cerebrospinal fluid lymphocytosis may be considered to indicate brainstem encephalitis under limited conditions. Most of our patients demonstrated clinical manifestations of myoclonus (963 cases, accounting for 85.6% of the total cases), of which 559 cases showed myoclonus alone (49.7%). In addition, of the 305 patients with routine lumbar puncture, 196 cases (17.4%) showed cerebrospinal fluid (CSF) lymphocytosis. The majority of these cases had good prognoses, and there was no significant correlation between myoclonus/CSF lymphocytosis and cardiopulmonary failure. By contrast, there were 158 cases (14.0%) of brainstem encephalitis of which 60.8% developed cardiopulmonary failure. Therefore, the misdiagnosis of myoclonus alone as brain stem encephalitis may interfere with determining patient prognosis. Because the present study was a retrospective analysis, subtle signs and symptoms were not recorded; therefore, we could not accurately estimate the myoclonic frequency, location, and time, which should be improved in future clinical and research work. However, if HFMD paediatric patients present with myoclonus alone and do not exhibit other nervous system damage or manifestations of ANS dysfunction, these patients should have a good prognosis. This is particularly significant in China. With the rapid economic growth of China and improvements in the standard of living, increasing numbers of middle class families demand optimal care. Moreover, because of concerns of the poor health services of rural hospitals, most of the patients brought their children to an urban hospital, leading to the urban hospital overwhelmed. At the peak of the HFMD pandemic, there were insufficient hospital beds for all of the patients who required hospitalization. Hence, it is very important to rapidly predict the prognosis of patients and reserve beds for paediatric patients with cardiopulmonary failure.

The present study confirmed that brainstem encephalitis alone, particular neurological complications (brainstem encephalitis with encephalitis, brainstem encephalitis with meningitis), and the number of CNS-involved regions were significant risk factors for cardiopulmonary failure. The reticular activating system (RAS) originates in the upper brainstem reticular core; therefore, when the brainstem is affected, the RAS is damaged, leading to autonomic dysfunction and cardiopulmonary failure^26^. A greater number of CNS-involved regions indicates a more extensive range of viral invasion and more cardiopulmonary failure. Therefore, if paediatric patients present neurological symptoms and signs of damage to the brainstem and cerebellum, such as cranial nerve damage, crossed paralysis or crossed sensory disturbances, ataxia, nystagmus, or changes in brainstem MRI imaging, close monitoring should be conducted, and interventions should be implemented as early as possible.

Encephalitis alone may not have been a risk factor because the Guide^14^ defines seizures as a symptom of encephalitis. However, the seizures are more likely to be simple febrile seizures^2^,^40^ and not a symptom of encephalitis if they occur in children 6 to 60 months old and are accompanied by fever (temperature ≥38.0 °C) and if the patient quickly regains consciousness. These patients have a good prognosis because only 1% of febrile seizures will lead to epilepsy^41^, and this type of seizure is often misdiagnosed as encephalitis and over-treated. Aseptic meningitis also had a good prognosis, which is consistent with previous reports^42^. In the present study, AFP alone (four cases) was not identified as a risk factor, which may be because the fact that the number of AFP cases in our study was small. Specifically, our study included 50 AFP cases of which 46 cases were combined with other neurological complications. Brainstem encephalitis combined with AFP, encephalitis combined with AFP and meningitis combined with AFP were risk factors for cardiopulmonary failure, and the univariate logistic regression analysis of the relationship between AFP (simple and mixed) and cardiopulmonary failure showed that AFP remained a risk factor for cardiopulmonary failure (odds ratio 10.9[6.0–19.8], *P* = 0.000).

EV-A71-related severe HFMD presents high rates of mortality and sequelae because of the lack of specific antiviral drugs. If young HFMD paediatric patients with a fever duration ≥3 days present neurological symptoms, a neurological examination should be carefully conducted, and the locations of the impaired regions of the nervous system should be quickly and accurately determined; if necessary, help from a neurologist may be required. For cases showing only myoclonus or only simple febrile seizures without other CNS or ANS symptoms, the patients can be followed at outpatient clinics or emergency rooms to avoid excessive medical treatment. If impaired consciousness or limb weakness occurs, if neurological symptoms and signs of brainstem and cerebellum involvement are present, or if multiple regions of the nervous system are affected, the patients should receive immediate intensive care and complete examinations, such as an ANS evaluation (respiration, heart rates, blood pressure, blood glucose monitoring, and CRT) and CRP, blood testing, cardiopulmonary functional detection, MRI, and lumbar puncture in a timely manner. Moreover, intervention should be conducted as early as possible to reduce the mortality and sequelae rates of EV-A71-related HFMD. However, the methods used in this study may not be the most optimal without high sensitivity and we should be cautious to apply it. In addition, the treatment for patients may be a confounder and should be considered in the future.

## Additional Information

**How to cite this article**: Long, L. *et al.* Neurological complications and risk factors of cardiopulmonary failure of EV-A71-related hand, foot and mouth disease. *Sci. Rep.* **6**, 23444; doi: 10.1038/srep23444 (2016).

## Supplementary Material

Supplementary Information

## Figures and Tables

**Table 1:** Detailed numbers of cases showing each type of neurological complication associated with EV-A71-related HFMD.

	Myoclonus alone	Simple neurological complications (n = 238, 23.40%)				Combination of two neurological complications (n = 158, 15.5%)						Combination of three neurological complications (n = 53, 5.2%)				Combination of four neurological complications
		Brainstem encephalitis alone	Encephalitis alone	Meningitis alone	AFP alone	Brainstem encephalitis combined with encephalitis	Brainstem encephalitis combined with meningitis	Brainstem encephalitis combined with AFP	Encephalo-meningitis	Encephalitis combined with AFP	Meningitis combined with AFP	Only without brainstem encephalitis	Only without encephalitis	Only without meningitis	Only without AFP	
Number of patients	559	35	109	90	4	37	30	5	71	10	5	11	1	5	36	9
Percentage of total neurological complications	54.97%	3.44%	10.72%	8.85%	0.39%	3.64%	2.95%	0.49%	6.98%	0.98%	0.49%	1.08%	0.10%	0.49%	3.4%	0.88%

AFP: Acute flaccid paralysis.

**Table 2:** Characteristics of 1,125 EV-A71-related HFMD patients.

	Simple HFMD (n = 95)	CNS Involvement (n = 1017)	ANS Involvement (n = 247)	Cardiopulmonary failure (n = 149)
Sex(male/female)	56/39	647/370	158/89	99/50
Mean(SD)age(months)	26.2(22.6)	25.8(15.5)	24.3(13.1)	20.6(10.2)
Mean(SD) length of stay (days)	5.8(2.8)	8.3(9.9)	10.7(10.1)	15.1(22.1)

CNS: central nervous system; ANS: autonomic nervous system.

**Table 3:** Adjusted OR of risk factors and neurological complications significantly associated with cardiopulmonary failure infected by EV-A71 in multivariate logistic regression model.

Risk Factors and Neurological complications	Adjusted Odds ratio (95% CI)	*P*
Risk Factors		
Age^Ψ^		0.000
X_1_	9.3(3.6–23.8)	0.000
X_2_	5.0(1.9–13.3)	0.001
X_3_	5.6(2.1–15.0)	0.001
Fever duration ≥3 days	9.4(1.4–62.5)	0.020
Coma	27.7(5.3–145.4)	0.000
Limb weakness	5.0(2.6–9.6)	0.000
Drowsiness	4.1(2.5–6.9)	0.000
ANS involvement	13.5(8.2–22.3)	0.000
Neurological complications		
Brainstem encephalitis alone	9.7(4.3–22.1)	0.000
Brainstem encephalitis combined with encephalitis	10.5(4.2–26.0)	0.000
Brainstem encephalitis combined with meningitis	2.3(1.0–5.2)	0.056
Number of CNS-involved regions	6.3(4.6–8.6)	0.000

^*^In the logistic model of risk factors, the sample size of the validation subset was 1,065 cases, whereas in the model of neurological complications, the sample size of the validation subset was 960 cases (excluding the simple HFMD cases).

^Ψ^X_1_, X_2_, and X_3_ were dummy variables for the age groups (0–1.5, 1.5–2, 2–3 and 3+ years). We used the 3+ years group as the reference group, X_1_ represented the 0–1.5 years group, X_2_ represented the 1.5–2 years group, and X_3_ represented the 2–3 years group.

ANS: Autonomic nervous system. CNS: Central nervous system.
