# Supplementary material for: Neurological complications and risk factors of cardiopulmonary failure of EV-A71-related hand, foot and mouth disease
Source: Sci Rep. 2016 Mar 22;6:23444. doi: 10.1038/srep23444 (PMC4802311; doi:10.1038/srep23444)
Supplement: Supplementary Information [file srep23444-s1.doc]

**Neurological complications and risk factors of cardiopulmonary failure of EV-A71-related hand, foot and mouth disease: Supplementary Tables**

**Lili Long ^1†^, Lin Xu ^1,2†^, Zhenghui Xiao ^3^, Shixiong Hu ^4^, Ruping Luo ^5^, Hua Wang ^6^, Xiulan Lu ^7^, Zhiyue Xu ^8^, Xu Yao ^9^, Luo Zhou ^1^, Hongyu Long ^1^, Jiaoe Gong ^9^, Yanmin Song ^1^, Li Zhao ^10^, Kaiwei Luo ^4^, Mengqi Zhang ^1^, Li Feng ^1^, Liming Yang ^9^, Xiaoqi Sheng ^11^, Xuegong Fan ^12^ and Bo Xiao ^1*^**

*1 Department of Neurology, Xiangya Hospital, Central South University, Changsha, China, 410008*

*2 General Hospital, Tianjin Medical University, Department of Neurology, Tianjin, China, 300052*

*3 Emergency center, Hunan Children's Hospital, Changsha, China, 410007*

*4 Hunan Provincial Center for Disease Control and Prevention, Changsha, China, 410005*

*5 Department of Infectious disease, Hunan Children's Hospital, Changsha, China, 410007*

*6 Department of Genetics, The Maternity and Child Health Hospital of Hunan Province, Changsha, China, 410008*

*7 Department of Intensive Care Unit, Hunan Children's Hospital, Changsha, China, 410007*

*8 Hunan Children's Hospital, Changsha, China, 410007*

*9 Department of Neurology, Hunan Children's Hospital, Changsha, China, 410007*

*10 Medical Records Management and Information Statistics Office, Xiangya Hospital, Central South University, Changsha, China, 410008*

*11 The Maternity and Child Health Hospital of Hunan Province, China, 410008*

*12 Department of Infectious disease, Xiangya Hospital, Central South University, Changsha, China, 410008*

†These authors contributed equally to this work.

*Corresponding author. Email: xiaobo1962_xy@163.com

**Table S1. The indicators for** heart rate, respiration and blood pressure

| **Vital signs** | **Increase** | **Criteria** |
| --- | --- | --- |
| Blood pressure(BP) | [Hypertension](../../../../D:/Desktop/%E5%A4%A7%E4%BF%AE3/%E5%A4%A7%E4%BF%AE2/20150920/%E5%A4%A7%E4%BF%AE1/20150507/%E6%89%8B%E8%B6%B3%E5%8F%A3%E6%B1%87%E6%80%BB/%E5%AE%9A%E4%BD%9C%E8%80%85%E5%90%8E/fujian/%E4%BD%93%E5%BE%81.xlsx#RANGE!A13) | age＜7years: BP≥120/80 mmHg |
|  |  | age≥7years: BP≥130/90 mmHg |
| Respiration(R) | [Dyspnea](../../../../D:/Desktop/%E5%A4%A7%E4%BF%AE3/%E5%A4%A7%E4%BF%AE2/20150920/%E5%A4%A7%E4%BF%AE1/20150507/%E6%89%8B%E8%B6%B3%E5%8F%A3%E6%B1%87%E6%80%BB/%E5%AE%9A%E4%BD%9C%E8%80%85%E5%90%8E/fujian/%E4%BD%93%E5%BE%81.xlsx#RANGE!A14) | age＜2months: R≥60 times/minute |
|  |  | 2 months≤age＜1 year: R≥50 times/minute |
|  |  | 1 year≤age＜5 years: R≥40 times/minute |
|  |  | age≥5 years: R≥24 times/minute |
| Heart rate(HR) | [Tachycardia](../../../../D:/Desktop/%E5%A4%A7%E4%BF%AE3/%E5%A4%A7%E4%BF%AE2/20150920/%E5%A4%A7%E4%BF%AE1/20150507/%E6%89%8B%E8%B6%B3%E5%8F%A3%E6%B1%87%E6%80%BB/%E5%AE%9A%E4%BD%9C%E8%80%85%E5%90%8E/fujian/%E4%BD%93%E5%BE%81.xlsx#RANGE!A15) | age＜2 years: HR≥180 bpm |
|  |  | 2 years ≤ age ＜6 years: HR≥160 bpm |
|  |  | 6 years ≤ age ＜14 years: HR≥120 bpm |

**Table S2.** The reference value of the blood lymphocyte Subsets in different age

| **Age** | **CD3 T cell(fifth to 95th percentiles)** | **CD4 T cell(fifth to 95th percentiles)** | **CD8 T cell(fifth to 95th percentiles)** |
| --- | --- | --- | --- |
| **2-3 months** | 60-87% | 41-64% | 16-35% |
| **4-8 months** | 57-84% | 36-61% | 16-34% |
| **12-23 months** | 53-81% | 31-54% | 16-38% |
| **24-59 months** | 62-80% | 35-51% | 22-38% |

**Table S3. The detail numbers of cases showing each type of neurological complication every month**

| **Month** | **brain stem encephalitis**  **(percentage of cases/month)** | **encephalitis**  **(percentage of cases/month)** | **meningitis**  **(percentage of cases/month)** | **AFP**  **(percentage of cases/month)** | **cases/month** |
| --- | --- | --- | --- | --- | --- |
| **Jan** | 0, 0.0% | 3, 100.0% | 0, 0.0% | 0, 0.0% | 3 |
| **Feb** | 1, 33.3% | 10.33.0% | 0, 0.0% | 1, 33.3% | 3 |
| **Mar** | 2, 25.0% | 4, 50.0% | 1, 12.5% | 1, 12.5% | 8 |
| **Apr** | 17, 27.4% | 26, 41.9% | 16, 25.8% | 3, 4.8% | 62 |
| **May** | 35,22.9% | 55, 36.0% | 54, 35.3% | 9, 5.9% | 153 |
| **Jun** | 59, 20.6% | 101, 35.2% | 106, 36.9% | 21, 7.3% | 287 |
| **Jul** | 15, 19.2% | 34, 43.6% | 22, 28.2% | 7, 9.0% | 78 |
| **Aug** | 6, 16.7% | 13, 36.1% | 14, 38.9% | 3, 8.3% | 36 |
| **Sep** | 6, 16.2% | 15, 40.5% | 15, 40.5% | 1, 2.7% | 37 |
| **Oct** | 12, 28.6% | 15, 35.7% | 13, 31.0% | 2, 4.8% | 42 |
| **Nov** | 2,9.1% | 13, 59.1% | 6, 27.3% | 1, 4.6% | 22 |
| **Dec** | 3, 15.8% | 9, 47.4% | 6, 31.6% | 1, 5.3% | 19 |

**Table S4. Factors significantly associated with cardiopulmonary failure HFMD cases infected by EV-A71**

| **Risk Factors** | **Non-Cardiopulmonary failure (n=928)** | **Cardiopulmonary failure (n=137)** | **Unadjusted**  **Odds ratio (95%CI)** | **P** |
| --- | --- | --- | --- | --- |
| **Male** | 578(62.3%) | 93(67.9%) | 0.8(0.5-1.1) | 0.206 |
| **Age^Ψ^** |  |  |  | 0.001 |
| **X_1_**  **X_2_**  **X_3_** |  |  | 3.9(2.0-7.6)  2.6(1.3-5.3)  2.7(1.3-5.5) | 0.000  0.008  0.006 |
| **PBT^*^≥38·5℃** | 811(89.3%) | 125(91.2%) | 1.2(0.7-2.3) | 0.493 |
| **Fever duration≥3 days** | 824(89.0%) | 135(98.5%) | 8.4(2.0-34.3) | 0.003 |
| **Tachypnea** | 119(12.9%) | 71(52.6%) | 7.5(5.1-11.1) | 0.000 |
| **Tachycardia** | 15(1.6%) | 39(28.7%) | 24.3(12.9-45.8) | 0.000 |
| **Hypertension** | 48(5.4%) | 57(42.5%) | 13.0(8.3-20.3) | 0.000 |
| **CRT＞2s** | 107(11.5%) | 71(51.8%) | 8.3(5.6-12.2) | 0.000 |
| **Mycoplasma infection** | 157(35.4%) | 22(31.9%) | 0.9(0.5-1.5) | 0.565 |
| **Hyperglycemia** | 56(17.0%) | 75(68.8%) | 10.8(6.5-17.7) | 0.000 |
| **Leucocytosis** | 54(6.1%) | 17(12.7%) | 2.2(1.3-4.0) | 0.006 |
| **CRP≥40 mg/L** | 15(2.4%) | 8(8.2%) | 3.6(1.5-8.7) | 0.005 |
| **Lymphocyte subsets abnormal** | 92(56.4%) | 39(60.0%) | 1.2(0.6-2.1) | 0.624 |
| **Myoclonus** | 786(84.7%) | 123(89.8%) | 1.6(0.9-2.8) | 0.119 |
| **Seizure** | 32(3.4%) | 7(5.1%) | 1.5(0.7-3.5) | 0.337 |
| **Vomiting** | 288(31.0%) | 78(56.9%) | 2.9(2.0-4.2) | 0.000 |
| **Coma** | 2(0.2%) | 19(13.9%) | 74.6(17.1-324.1) | 0.000 |
| **Limb weakness** | 37(4.0%) | 44(32.1%) | 11.4(7.0-18.5) | 0.000 |
| **Headache** | 30(3.2%) | 7(5.1%) | 1.6(0.7-3.7) | 0.267 |
| **Drowsiness** | 120(12.9%) | 72(52.6%) | 7.5(5.1-11.0) | 0.000 |
| **CSF-WBC^*^≥10*10^6^/L** | 130(61.9%) | 60(69.0%) | 1.3(0.8-2.2) | 0.304 |
| **Intracranial hypertension** | 112(54.4%) | 58(61.7%) | 1.4(0.8-2.2) | 0.235 |
| **ANS involvement** | 127(13.7%) | 105(76.6%) | 20.7(13.4-32.1) | 0.000 |

PBT: peak body temperature; CRT: Capillary refill time; CRP: C-reactive protein; CSF-WBC: cerebrospinal fluid- white blood cell.

ΨX1, X2, and X3 were dummy variables for the age groups (0-1.5, 1.5-2, 2-3 and 3+ years). We used the 3+ years group as the reference group, X1 represented the 0-1.5 years group, X2 represented the 1.5-2 years group, and X3 represented the 2-3 years group.

**Table S5. Neurological complications significantly associated with cardiopulmonary failure HFMD cases** infected by EV-A71

| **Neurological complications** | **Non-** **Cardiopulmonary failure**  **(n=825)** | **Cardiopulmonary failure (n=135)** | **Unadjusted**  **Odds ratio** | **P** |
| --- | --- | --- | --- | --- |
| **Male** | 517(62.7%) | 92(68.1%) | 0.8(0.5-1.2) | 0.221 |
| **Ages^Ψ^** | - | - | - | 0.001 |
| **X_1_**  **X_2_**  **X_3_** | -  -  - | -  -  - | 3.9(2.0-7.6)  2.6(1.3-5.2)  2.6(1.3-5.3) | 0.000  0.009  0.010 |
| **Myoclonus alone** | 523(63.4%) | 6(4.4%) | 0.03(0.01-0.06) | 0.000 |
| **Brain stem encephalitis alone** | 20(2.4%) | 13(9.6%) | 4.3(2.1-8.8) | 0.000 |
| **Encephalitis alone** | 92(11.2%) | 6(4.4%) | 0.4(0.2-0.9) | 0.022 |
| **Meningitis alone** | 81(9.8%) | 5(3.7%) | 0.4(0.1-0.9) | 0.027 |
| **AFP alone** | 4(0.5%) | 0(0.0%) | infinite | 1.000§ |
| **Brainstem encephalitis combined with encephalitis** | 7(0.8%) | 27(20.0%) | 29.2(12·4-68·7) | 0.000 |
| **Brainstem encephalitis combined with meningitis** | 15(1.8%) | 13(9.6%) | 5.8(2.7-12.4) | 0.000 |
| **Brainstem encephalitis combined with AFP** | 2(0.2%) | 2(1.5%) | 6.2(0.9-44.3) | 0.070 |
| **Encephalomeningitis** | 56(6.8%) | 13(9.6%) | 1.5(0.8-2.8) | 0.238 |
| **Encephalitis combined with AFP** | 6(0.7%) | 4(3.0%) | 4.2(1.2-15.0) | 0.029 |
| **Meningitis combined with AFP** | 3(0.4%) | 2(1.5%) | 4.1(0.7-24.9) | 0.123 |
| **Number of CNS involved regions** | — | — | 5.9(4.5-7.7) | 0.000 |
| **1 region**  **2 regions**  **3 regions**  **4 regions** | 197(23.9%)  90(10.9%)  15(1.8%)  0(0.0%) | 24(17.8%)  61(45.2%)  35(25.9%)  9(6.7%) | —  —  —  — | —  —  —  — |

**P*=1.000 by Fisher's exact test

AFP: Acute flaccid paralysis. CNS: Central nervous system.

ΨX1, X2, and X3 were dummy variables for the age groups (0-1.5, 1.5-2, 2-3 and 3+ years). We used the 3+ years group as the reference group, X1 represented the 0-1.5 years group, X2 represented the 1.5-2 years group, and X3 represented the 2-3 years group.

The detail and usage of the model were provided below:

The first model: $P_{1}\text{=}\frac{\text{e}^{\left(-7.621+2.228X_{1}+1.614X_{2}+1.717X_{3}+3.320X_{4}+1.612X_{5}+1.421X_{6}+2.244X_{7}+2.605X_{8}\right)}}{1+\text{e}^{\left(-7.621+2.228X_{1}+1.614X_{2}+1.717X_{3}+3.320X_{4}+1.612X_{5}+1.421X_{6}+2.244X_{7}+2.605X_{8}\right)}}$

X_1_, X_2_, and X_3_ were dummy variables for the age groups (0-1.5, 1.5-2, 2-3 and 3+ years). We used the 3+ years group as the reference group, X_1_ represented the 0-1.5 years group, X_2_ represented the 1.5-2 years group, and X_3_ represented the 2-3 years group, X_4_ = coma, X_5_ = limb weakness, X_6_ = drowsiness, X_7_ = Fever duration≥3 days, X_8_ = ANS involvement, all the X are binary variables.

We picked up P_1_=0.100 as the cut-off. In other words, If P_1_ is greater than 0.100, there is a big chance that the patients would develop into cardiopulmonary failure.

For example, there is a 4 years old EV-A-71 related patients presented with coma, limb weakness, drowsiness, fever duration≥3 days, and ANS involvement, $P_{1}\text{=}\frac{\text{e}^{\left(-7.621+2.228*0+1.614*0+1.717*0+3.320*1+1.612*1+1.421*1+2.244*1+2.605X*1\right)}}{1+\text{e}^{\left(-7.621+2.228*0+1.614*0+1.717*0+3.320*1+1.612*1+1.421*1+2.244*1+2.605X*1\right)}}$≈0.973, the probability of the patient develop into cardiopulmonary failure is 0.973 ≥0.100. So the patient should receive immediate intensive care.

The second model: $P_{2}\text{=}\frac{\text{e}^{\left(-5.703+1.553X_{1}+1.104X_{2}+0.813X_{3}+2.277X_{4}+2.350X_{5}+0.811X_{6}+1.836X_{7}\right)}}{1+\text{e}^{\left(-5.703+1.553X_{1}+1.104X_{2}+0.813X_{3}+2.277X_{4}+2.350X_{5}+0.811X_{6}+1.836X_{7}\right)}}$

X_1_, X_2_, and X_3_ were dummy variables for the age groups (0-1.5, 1.5-2, 2-3 and 3+ years). We used the 3+ years group as the reference group. X_4_ = simple brainstem encephalitis, X_5_ = brainstem encephalitis combined with encephalitis, X_6_ = brainstem encephalitis combined with meningitis, X_7_= the number of CNS involved regions

If P_2_ is greater than 0.199, there is a big chance that the patients would develop into cardiopulmonary failure. The usage of the second model is similar to the first model.

**Reference**

1. Yi, Z. The classification of children glomerular disease in *Pediatrics 2nd edn.* (ed. Xue, X. D.). 324-326(Beijing 2010).
2. Heffelfinger, J. D. *et al*. Evaluation of children with recurrent pneumonia diagnosed by World Health Organization criteria. *Pediatr Infect Dis J* **21**, 108-112 (2002).
3. Gui, Y. Congestive heart-failure in *Pediatrics* 2nd edn. (ed. Xue, X. D.). 490-492 (Beijing 2010).
4. Denny, T. *et al*. Lymphocyte subsets in healthy children during the first 5 years of life. *JAMA* **267**, 1484-1488 (1992).
